# Measurement and Definitions of Obesity In Childhood and Adolescence: A field guide for the uninitiated

**DOI:** 10.1186/1475-2891-6-32

**Published:** 2007-10-26

**Authors:** Helen N Sweeting

**Affiliations:** 1 MRC Social and Public Health Sciences Unit, 4, Lilybank Gardens, Glasgow, G12 8RZ, UK

## Abstract

This paper aims to guide readers embarking on the complex literature in respect of childhood and adolescent obesity. It opens with a discussion of definitions of 'obesity' based on overall fat levels and the significance of fat distribution. This is followed by simple descriptions of the various techniques used to measure fat, including density-based, scanning, bioelectrical impedance and anthropometric methods. The paper then turns to 'overweight' and the measurement of weight in relation to height, particularly via body mass index (BMI). While it is a relatively simple measure and a valuable tool, BMI has several disadvantages, which are described. These include a lack of consensus on which values should be used to define 'overweight' or 'obese', with the result that the literature contains a confusing multiplicity of child and adolescent obesity rates.

## Introduction

At one level, research on child and adolescent obesity rates is easy to understand. Based on recent studies, the BBC News website includes statements such as 'obesity affects 12% of under-11s' (14^th ^December, 2006), and 'Levels of obesity in children aged two to 10 years rose from 9.9% to 13.4% between 1995 and 2004, according to the Health Survey for England.' (25^th ^January, 2007). However, for the researcher who wishes to gain a clearer understanding of how obesity is measured, or to delve into these figures in more detail, the literature may prove quite challenging.

One difficulty is that descriptions of the measurement of obesity are littered with acronyms, an understanding of which is often assumed. But for the uninitiated, what do ADP, DXA or BIA stand for? And perhaps even more puzzling, how is it possible to accurately measure levels of fat within a living individual? A second source of puzzlement is apparently conflicting numbers. For example, given that obesity levels are lower in the UK than the US [1], why would one paper report rates for US 12–19 year olds in 1999–2002 of 16%, but another report rates for English 11–15 year olds in 2000 of 18% [2,3]?

This paper is written by one who was puzzled, and is now less so, to help others embarking upon this literature. It begins with a discussion of the significance of overall fat levels and fat distribution, followed by a description of methods to measure fat. The second half of the paper describes measurements of overweight rather than fat. It focuses particularly on body mass index, since this is by far the most common indicator, exploring how it has been used to define obesity, and how accurately it identifies the fattest children. From this point forwards, terms commonly used in this literature are in bold the first time they appear.

## Obesity, fat levels and fat distribution

**Obesity **should be defined as excess body fat or adipose tissue; it is this, not weight which is associated with the comorbid conditions [4]. This being the case, the next question is what level of fat should be defined as 'obese'. Studies of children and adolescents which have examined the relationship between percentage of body fat calculated from skinfold measurements and indicators of biomedical status such as blood pressure and blood lipids, have suggested 30% fat in females and 20–25% in males [5,6]. There is also evidence of ethnic differences, for example, South Asian people appear to be sensitive to the metabolic consequences of obesity at lower levels than white people [7].

This is further complicated by findings that it is **central (also described as intra abdominal – IA, or visceral) fat **which is more pathogenic [4,8]. Adults with large waist circumferences have excess morbidity, including back pain, diabetes and CVD risk factors [9], and although less clear, there is some evidence of health risks associated with excess abdominal fat in children [10,11]. There is also evidence that the excess fat in obese children and adolescents is likely to accumulate in the abdominal regions [12,13].

Overall levels, as well as the distribution of fat, differ according to both sex and ethnicity. The **android (male, or 'apple shaped') fat pattern **is represented by relatively greater amounts in the upper body, the **gynoid (female, or 'pear') pattern **by greater amounts in the hip and thigh areas [14]. Female lower body fat is less metabolically active than that in the abdominal region, and is programmed to become mobilized during pregnancy and lactation. In relation to the greater pathogenicity of abdominal fat, it is interesting that mortality rates are higher among females with android fat patterning [8]. Sex differences in fat levels have generally been considered to become manifest during puberty [15]. Thus, in samples followed up through adolescence, levels of fat are higher among females, and of fat-free mass among males [16-20]. However, more recent studies of pre-pubertal children, some as young as 3 years old, in the US, UK, Germany, Italy and China, have also found higher percentages of body fat and evidence of the gynoid pattern among females [21-25].

Percentage body fat also appears to be lower in black, perhaps particularly black African children (and adults) compared with Caucasians. In other words, for any given body mass, black African children have higher fat-free and lower fat mass. Levels of abdominal fat also tend to be lower among Black Africans. There is, in addition, some evidence that these differences are more pronounced among females than males [10,17,21,26]. In contrast, many Asian races, and possibly also Hispanics and Chinese, carry a higher percentage fat mass than Caucasians [4,25,26].

## Methods to measure fat

Table 1 describes the main techniques which have been adopted to measure fat in human subjects. They are categorized as density-based (hydrodensitometry; air displacement plethysmography), scanning (computerized tomography; magnetic resonance imaging; dual-energy x-ray absorptiometry), bioelectrical impedance and anthropometric (skinfold; waist circumference; waist-hip ratio) methods, according to the general principle on which they are based. More complex and detailed descriptions are available [27-29]. As Table 1 demonstrates, the majority of these methods are complex and limited to research settings.

**Table 1:** Methods to measure fat

	**General principle**	**Method**	**Acronym**	**Methodology**	**Method-specific principle**	**Further comments**
**Density-based methods**	If the density (weight per unit volume) of a human body is known, then the relative proportions of fat and fat-free mass can be estimated using an equation such as those of Siri [72] or Lohman [73]. While mass can be easily determined by weighing, volume measurements are more difficult [74].	Hydrodensitometry (underwater weighing)	UWW	Weighs the subject while submerged in a large tank (having exhaled maximally) and also outside the tank [29].	Based on Archimedes' principle (buoyancy law) that if the density of an object exceeds that of water, it will sink. Given two people of equivalent weight outside the tank, the one with more fat, which is less dense than water, will weight less in water than the one with more fat-free tissue (such as bone and muscle) which is more dense than water [75]. (In fact, it is unnecessary to actually weight the subject underwater, since their volume can also be assessed via the amount of water displaced when they are submerged.)	Often described as 'the gold standard', but time-consuming and requires the subject to submerge themselves, so particularly unsuitable for certain populations, such as children, and limited to research settings [76].
		Air Displacement Plethysmography	ADP	Measures the volume of air the subject displaces inside an enclosed chamber.	Given the subject's volume and weight, their density can be calculated.	Early plethysmographs were complex, inconvenient and required temperature-controlled surroundings. A simple, quick automated plethysmograph [77] has been available since mid 1990s, but is still limited to research settings [74].
**Scanning methods**	Can assess not just overall fat mass, but also its regional distribution.	Computerised Tomography; Magnetic Resonance Imaging	CT; MRI	CT – a series of x-rays pass through the body at different angles. MRI – uses a strong magnetic field and a radio wave antenna which sends signals to the body and then receives them back. These are used to produce internal images.	Both allow for the creation of cross-sectional high-resolution internal images.	Expensive, involve radiation exposure (CT) and limited to research settings [27-29].
		Dual-Energy X-ray Absorptiometry	DEXA or DXA	A series of transverse scans, via low energy x-ray beams, progress inch-by-inch across the body and are collected by an external detector.	The beams are differentially absorbed by the various different tissues (fat, bone, etc) in the body.	Can be used to calculate fat and fat-free mass, and both total and regional body composition in subjects over a wide range of ages and body sizes. Relatively low radiation dose. Validated against UWW and comparison with animal carcasses in the pediatric weight range. Use limited to research settings [27, 28, 46, 78, 79].
**Bioelectrical impedance methods**	Electric currents pass more easily through body fluids in muscle and blood, but encounter resistance ('biolectical impedance') when they pass thorugh fat, since it contains little water.	Bioelectrical Impedance Analysis	BIA	Conductors are attached to the subject's body, and a low, safe, current is sent through. Electrodes are generally placed at wrist and ankle; an increasingly commonly used analyser requires subjects to stand on it in bare feet and hold a handgrip in each hand. Foot-to-foot BIA measures the impedance of the lower body and only requires the subject to stand on pad electrodes.	The resistance between the conductors provides a measure of body fat.	Although less accurate than more sophisticated measurements, some current analysers are relatively inexpensive, portable, simple and quick, meaning BIA can now be used in the field and with large samples [20, 80].
**Anthropometric methods**	Direct measurements of various body parameters.	Skinfold measurements	SF	Subcutaneous (but not internal) fat is measured by firmly grasping a fold of skin with callipers and raising it, with no muscle included. Single site measurements, e.g. triceps skinfolds [16] are simplest. An alternative is to add skinfolds from a variety of sites, generally representing both peripheral and trunk areas [17].	Subcutaneous fat may be taken as an indicator of total fat. Fat distribution can also be determined via the ratio of trunk to peripheral skinfolds [81]. It is also possible to calculate total body fat via equations: Slaughter's equations predict percent body fat from the sum of triceps plus subscapular, or triceps plus calf in children and young people [82]; more recent equations by Dezenberg use triceps skinfolds plus body weight, sex and ethnicity [83].	Cheap and fairly simple, but the need to partially undress may put some subjects off, leading to bias. Also difficult to measure reproducibly, particularly if the subject is fat [84].
		Waist circumference	WC	Ideally measured using a flexible plastic tape with a sprung handle to ensure reproducible levels of tension [29]. Since a potential source of error is incorrectly positioning the tape, the measurement site is generally specified by reference to specific anatomic landmarks [85].	WC reflects total and abdominal fat levels, and as an indicator of adiposity is not greatly influenced by height [86].	WC centiles for children have been developed in a number of countries [86-89]. It has also recently been suggested that the ratio of waist to height could be used as a rapid screening tool [90].
		Waist-hip ratio	WHR		A larger WHR in adults indicates relatively larger amounts of abdominal fat and has been used to describe body fat distribution. However it is influenced by several other bodily factors and there is some evidence that it is a poorer measure of body fat distribution in children [91].	Infrequently used in studies of children and adolescents.

## When fat cannot be measured – weight in relation to height

In contrast to obesity, which is excess levels of fat, **overweight **is excess weight in relation to height, and can be easily measured using only a set of scales and a stadiometer. Indeed, it may not even require this; some studies have used self-report weights and heights. However, comparisons with measured values show that although correlations are high, weight tends to be under-reported, particularly among females and the overweight, while height may be over-reported. There is a lack of consensus on the impact that this has on measures of weight in relation to height, some authors suggesting that self-report values are adequate [30], others advocating caution [31,32].

### Measures of weight in relation to height

Although body weight, particularly at very high levels, tends to be associated with adiposity, weight alone is an insufficient measure of obesity, because it is correlated with height [33]. A number of measures of weight in relation to height have been devised. The simplest is **weight for height**. In 1977 a World Health Organization (WHO) bulletin noted that in undernourished populations, 80% median weight for height (which corresponds to approximately -2.0 standard deviations) was suitable for classifying malnourished children. Following this principle, it was suggested that 120% (or +2.0 standard deviations) could be used in populations where over-nutrition was a problem [34]. Although adopted in some recent studies [35,36], such measures tend not to be used currently. **Body Mass Index (BMI)**, defined as weight (kg)/height squared (m^2^) is the most frequently used measure of weight in relation to height, but there are others. These include **Rohrer's Ponderal Index (termed either Rohrer's Index – RI, or the Ponderal Index – PI)**, defined as weight/height^3^. This has been compared with BMI in respect of its ability to predict percentage body fat in children and adolescents, and its long-term associations with adult obesity [37,38]. Although it may perform as well or better in some respects than BMI, it is much less used with children and adolescents, although it remains popular with neonatologists [39]. Finally, there is **Benn's Index**, defined as weight/height^p ^where the power p is chosen so the index is independent of height. While this may be 'the ideal index' ([39], p.289), the fact that p is neither constant, nor necessarily a whole number, means the calculations are very complicated, and such indices are rarely used [40,41].

### Body Mass Index

As noted above, BMI is the most frequently used measure of weight in relation to height. It has been described as 'the backbone of the obesity classification system and surveillance statistics ... an immensely valuable tool' ([4], p.141). However, a number of authors have detailed its disadvantages [42-45].

The first of these is that BMI varies between males and females and according to age and level of maturity. Thus, while male and female BMIs tend to be similar in childhood, they are higher among females in adolescence. In respect of age, BMI increases from birth to around one year, then declines to around age six, then increases through the remainder of childhood and adolescence. The point at which BMI reaches its lowest level and begins to increase is termed **'adiposity rebound'**, with earlier adiposity rebound being associated with increased risk of subsequent overweight [46]. Such variations mean that among children and adolescents the significance of any particular BMI is more difficult to determine than within adult populations.

A second, and related limitation of BMI, is that it reflects both fat and fat-free components of body weight. However, as described earlier, populations differ in respect of both percentage fat mass and fat distribution, and in the relation between body composition and morbidity. This means, again, that the significance of any particular BMI will vary. Thus, among children with the same BMI, fat measurements are higher for whites than for blacks [47]. Further, recent studies have suggested that increases in overall BMIs have been accompanied by larger increases in the percentage as fat mass and concomitant decreases in fat free mass (attributed to reduced activity levels). Importantly, this suggests that recent increases in *adiposity *are even greater than those suggested by increases in BMI [13,23,48].

A third disadvantage of BMI is that since one of its components is height, the index also varies according to height, and this association in turn varies according to sex and age. Its relationship to height means that BMI is also affected by relative leg length.

A further disadvantage is that since BMI does not measure fat directly, there is no consensus about which cut-off to use in order to define obesity in children and adolescents. Among adults, a WHO Expert Committee on Physical Status agreed, in 1993, to classify BMIs as follows: 25–29.9 as 'overweight' (or 'pre-obese'); 30–34.9 as 'obese class I'; 35–39.9 as 'obese class II'; and over 40 as 'obese class III'. This classification was based on the risk of comorbidities, rising across the four categories from 'increased', to 'moderate', 'severe' and 'very severe' [49,50]. Unfortunately, much less is known about levels of risk associated with specific BMI levels in children and adolescents. This means that statistical approaches have often been used. These involve working out the distribution for a particular population and rather arbitrarily choosing particular values – often the 85^th ^or 95^th ^centiles, which distinguish those with the highest BMIs from the rest of the population [33]. Since the distribution of BMI varies according to sex, age and ethnicity, sex- and age- (and, sometimes, in the US, race-) specific centile curves are calculated.

### BMI and overweight/obesity – where to draw the line?

Clearly, if the aim is to track levels of obesity over time, or to compare populations, the BMI centile values defining obesity must be fixed. The question is not only which centile they should be fixed at, but also which population (and so which point in time) should be used as the basis for the calculations?

Within the US, reference growth charts based on nationally representative surveys (the **National Health Examination Surveys – NHES**, later called the **National Health and Nutrition Examination Surveys – NHANES**) have been produced since 1977 [51], and in 1991, race-specific and population-based BMI centiles, covering ages 6–74 were generated. These are often described as the **MDD definitions**, based on the initial letters of the authors' surnames [52]. An expert committee recommended their use for children and adolescents, with the 95^th ^BMI centile for age and sex (or BMI 30 kg/m^2^) as cut-offs for 'overweight', and the 85^th ^centile as 'at risk of overweight' for screening purposes. The fact that the committee decided not to use the term 'obese' (which they associated with excess fat rather than weight) [53] has lead to some confusion [33]. The most recent US charts were produced by the Centers for Disease Control in 2000 (hence termed **'CDC 2000'**). The inclusion of more recent data would have shifted the weight and BMI curves upwards, resulting in a high proportion of fatter children being characterized as 'normal'. In order to avoid this, data obtained since 1988 from those aged over 6 years were excluded [54]. These charts also tend to be used within Canada and Australia.

Within the UK, charts (**'UK90'**) have been produced, based on data from several surveys, conducted 1978–90 and including around 30,000 subjects. Although the authors suggested the 98^th ^centile, which at age 20 is 29.0 kg/m^2 ^(thus close to the adult definition of 30 kg/m^2^) as 'a reasonable definition of child obesity' ([55], p.28), the 95^th ^centile is more commonly adopted for epidemiological purposes. Similar BMI-for-age sex-specific reference charts have been developed in several other European countries including France, Germany and Denmark [56-58].

Against this background, the **International Obesity Task Force (IOTF)**, a global network of expertise, concluded that the definition of obesity in children and adolescents should be consistent with that for adults, and that, ideally, it should be based on a reference representative of the world's population [59]. Subsequently, data from 6 countries, collected 1963–93, was pooled, and in 2000, centile curves were published that pass through the points of 25 kg/m^2 ^and 30 kg/m^2 ^(reflecting WHO recommended definitions of adult overweight and obese) at age 18 [60]. In fact, the available reference data do not adequately represent the world's population. Further, as noted earlier, ethnic differences in body composition and the percentage of body fat associated with adverse health consequences mean that a single international definition of obesity may not be appropriate [44]. However, it has been suggested that despite these limitations, prevalence studies should present results based on the IOTF cut-offs as well as national definitions, since this would allow for comparison across populations [61].

The confusing multiplicity of child and adolescent obesity rates seen in the literature results from the use of different definitions; a number of studies have demonstrated this by applying several definitions to the same population. For example, one US study found rates of 13%, 11% and 8% when applying the MDD 'overweight', CDC 2000 'overweight' and IOTF 'obesity' criteria to (1998–1994) data in respect of 6–8 year old males [62]. Similarly, a UK study of 4–11 year olds (1994 data) found male and female obesity rates of 1.7% and 2.6% when applying the IOTF definition, but of 2.5% and 2.2% when applying the UK90 definition. Thus, while the IOTF definition suggested higher rates among females, the reverse was suggested by the UK90 definition [63].

Crucially, given that BMI measures excess weight rather than fat, studies have also examined the accuracy with which it identifies the fattest children and adolescents. This translates into its sensitivity (how good it is at identifying the truly obese) and specificity (how good it is at identifying the non-obese). Ideally, this requires a definition of 'true' obesity, however, as described earlier, this is lacking. Studies have therefore adopted a variety of methods. These include correlating BMI with percentage body fat (measured in various ways); comparing BMI-defined obesity (various different cutoffs) with obesity defined in terms of percentage body fat (also various different cutoffs, generally around 30% for females and 20–25% for males – see earlier and [5,6]); comparing BMI-defined obesity with percent body fat levels deemed 'high' (typically 85^th ^or 95^th ^percentile); and finally, comparing cardiovascular risk factors among those defined via BMI as obese and non-obese [64-67]. The consensus from such studies is that BMI is a reasonable measure of adiposity, although the relationship differs not only according to age, sex and ethnicity, but also degree of fatness, being better at high levels. This means that it is of more use in epidemiological studies, but is relatively poor at predicting fat mass in any individual child. Further, current BMI cutoffs have relatively high specificity, but lower sensitivity. This means that non-obese children are unlikely to be wrongly labeled, however obese children may be missed (for example, see [22,68-70]).

## Conclusion

Increasing rates – regardless of definition – have highlighted the importance of the measurement of child and adolescent obesity on a population level. However 'obesity' is a slippery concept. Even the WHO definitions of 'overweight' and 'obesity' adopted for use with adults are based on BMI levels associated with raised risks of comorbidities. However, 25 kg/m^2 ^and 30 kg/m^2 ^are also easily remembered, neat and tidy numbers. Other definitions would have been possible. In fact, lower cut-offs have subsequently been suggested for Asians, given their tendency to both carry a higher percentage fat and be more sensitive to its metabolic consequences [71].

Decisions in respect of children are even more complex. As with adults, the relationships between BMI and levels of fat, and between levels of fat and associated morbidities, differ between ethnic groups. In addition, BMI levels, and their relationship with levels of fat vary according to sex, age and maturity. This has resulted in attempts to define obesity on the basis of percentage body fat, waist circumference and, most frequently, BMI, which reflects weight in relation to height. The consequence of this is that different studies may define obesity in different ways. None could be said to be 'correct' [62]. It has been argued that efforts to assess body fat and develop population standards against which individuals can be compared should be increased [4]. However, even this would require decisions as to cutoffs.

The literature in this area can be confusing. While that seems unlikely to change in the near future, this guide may increase the understanding of those embarking upon it.
